# Association of Endothelin-1 rs5370 G>T gene polymorphism with the risk of nephrotic syndrome in children

**DOI:** 10.15171/jnp.2017.24

**Published:** 2016-12-17

**Authors:** Mohammad Hashemi, Simin Sadeghi-Bojd, Soheib Aryanezhad, Maryam Rezaei

**Affiliations:** ^1^Cellular and Molecular Research Center, Zahedan University of Medical Sciences, Zahedan, Iran; ^2^Department of Clinical Biochemistry, School of Medicine, Zahedan University of Medical Sciences, Zahedan, Iran; ^3^Children and Adolescents Health Research Center, Zahedan University of Medical Sciences, Zahedan, Iran; ^4^Department of Pediatrics, School of Medicine, Zahedan University of Medical Sciences, Zahedan, Iran

**Keywords:** Nephrotic syndrome, Endothelin-1, Gene polymorphism

## Abstract

**Background:**

Primary nephrotic syndrome (NS) is a common kidney disease in children. Objectives: The present study was aimed to investigate whether rs5370 G>T (lys198Asn) genetic variant of endothelin-1 (ET-1) is involved in the susceptibility to NS.

**Patients and Methods:**

This case-control study was performed on 138 patients with NS and 150 healthy children. Genomic DNA was extracted from whole blood using salting out method. Polymorphism of the ET-1 rs5370 G>T (lys198Asn) polymorphism detected by T-ARMS-PCR as well as PCR-RFLP method.

**Results:**

The results showed that the genotype and allelic frequencies of the ET-1 rs5370 G>T variant were not significantly different between cases and controls. Furthermore, subgroup analysis showed that rs5370 G>T variant was not associated with gender of patients. In NS patients the genotype was not associated with cholesterol, triglyceride, total protein and albumin levels.

**Conclusions:**

In conclusion, our findings indicate that ET-1 rs5370 G>T is not associated with NS. Further studies with larger sample sizes and different ethnicities are required to validate our findings.

**Implication for health policy/practice/research/medical education:**

In the present study we aimed to examine the impact of endothelin-1 (ET-1) rs5370 polymorphism on nephrotic syndrome (NS) in children. Our finding did not support an association between rs5370 variant and risk of NS in our population. In the forthcoming studies, other variants of endothelin-1 should be tested to find out the possible association between endothelin-1 polymorphisms and risk of NS.

## 1. Background

Nephrotic syndrome (NS) is a chronic pediatric disorder characterized by heavily proteinuria, hyperlipidemia, hypoalbuminemia, and peripheral edema (1,2). The annual incidence of NS is approximately 2 to 7 children per 100 000 (1). It has been proposed that minimal change NS (MCNS) and focal segmental glomerulosclerosis (FSGS) are the most common causes of idiopathic NS in children (3).

ET-1 is encoded by the *EDN1* gene which is located in chromosome 6 (6p21–24), and is a potent vasoconstrictor that acts as a modulator of vasomotor tone and vascular remodeling (4,5). Transgenic animal models in which the human ET-1 gene was transferred into the germline of mice were established independently by two research groups (6,7). The overexpression of ET-1 was associated with a pathological phenotype manifested by signs such as age-dependent development of renal cysts, interstitial fibrosis of the kidneys, and glomerulosclerosis leading to a progressive decrease in glomerular filtration rate (GFR). But the blood pressure was not affected even after the development of an impaired GFR. This finding was not in agreement with previous studies which showed that ET-1 is a potent vasoconstrictor when administered intravenously (6,8).

Previous studies showed an association between the coding polymorphism lys198Asn (rs5370 G/T) of ET-1 gene and hypertension, systolic blood pressure and HDL levels but the data were inconsistent (9-13). Polymorphisms in the ET-1 gene have been shown to be associated with the morbidity of IgA nephropathy (14) and the progression of autosomal dominant polycystic kidney diseases (15). There is little data concerning the impact of ET-1 variants on NS in children (16). To the bet of our knowledge, ET-1 polymorphisms were not investigated in NS in Iranian patients before.

## 2. Objectives

The present study aimed to examine the possible association between ET-1 rs5370 G>T gene polymorphism and NS in a sample of Iranian children.

## 3. Patients and Methods

This case-control study was conducted on 138 patients who suffering from NS in pediatric department of Ali-ebneh Abitaleb hospital, Zahedan, Iran (17). The control group consisted of 150 healthy children. All participants were examined by the same pediatric nephrologist. The characteristic of cases and controls are shown in Table 1. Genomic DNA was extracted from whole blood as described previously (18). The samples were stored at -20ºC until analysis.

Genotyping of ET-1 rs5370 G/T polymorphism was done using tetra amplification refractory mutation system-polymerase chain reaction (T-ARMS-PCR), which is a cost-effective and fast method for detection of single nucleotide polymorphism (19). We used four primers, two external primers (forward outer: 5′-CAGATTCAGGTTTTGTTTGTGCCAGATT-3′, reverse outer: 5′-TTGGGGGAACTCCTTAACCTTTCTTG-3′) and two allele-specific internal primers (forward inner [G allele]: 5′-ATGATCCCAAGCTGAAAGGCGAG-3′, reverse inner [T allele]: 5′-GGGTCACATAACGCTCTCTGGAGAGA-3′). Product sizes were 191 bp for T allele, 242 bp for G allele, and 385 bp for the two outer primers (control band).

**Figure 1:**
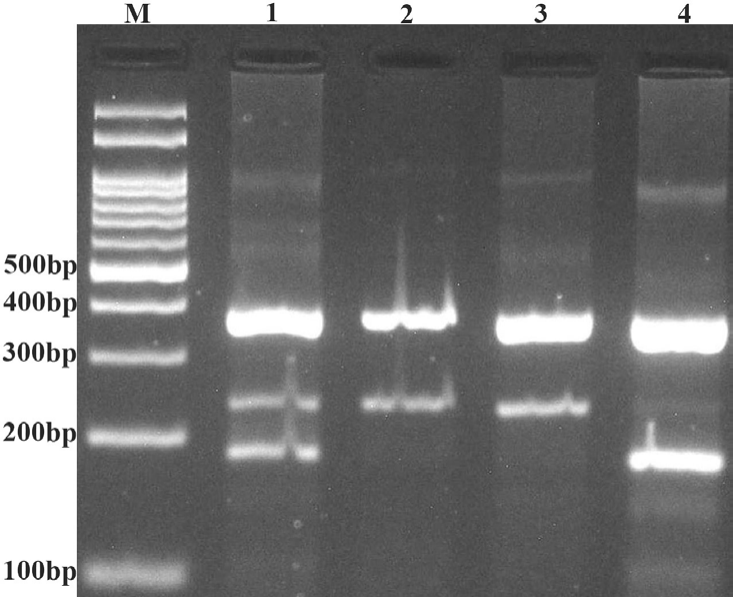
Photograph of the PCR product of the ET-1 rs5370 G>T polymorphism by T-ARMS-PCR method. M: DNA marker; Lane 1: GT; Lanes 2 and 3: GG; Lane 4: TT.

The PCR cycling condition were 5 minutes at 95°C followed by 30 cycles of 30 seconds at 95°C, 30 seconds at 55°C, and 30 seconds at 72°C with a final step at 72°C for 10 minutes to allow for complete extension of all PCR fragments. The PCR products were analyzed by electrophoresis on a 2.5% agarose gel containing 0.5 µg/mL ethidium bromide and visualized by transillumination with UV light; a photograph was then taken (Figure 1).

**Figure 2:**
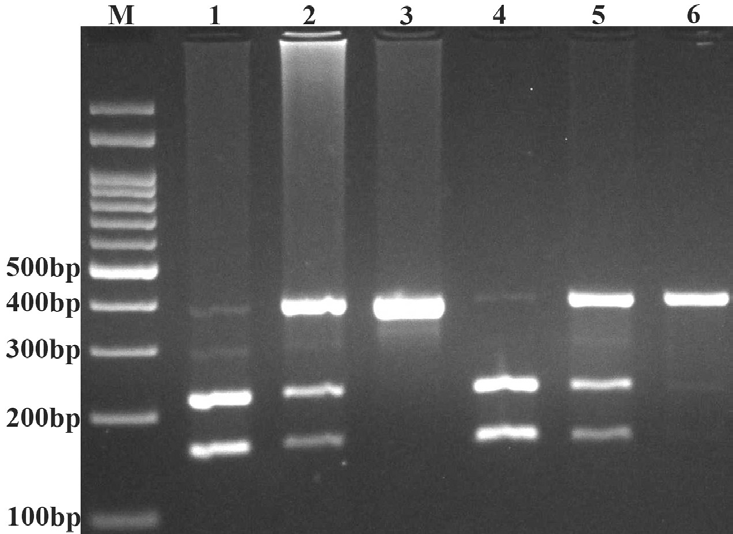
Photograph of the PCR product of the ET-1 rs5370 G>T by PCR-RFLP method. M: DNA marker; Lane 1 and 4: GG; Lanes 2 and 5: GT; Lanes 3 and 6: TT.

In addition, using forward and reverse primers of T-ARMS-PCR assay, we performed PCR-RFLP. Ten microliter of PCR product digested by Cac8I restriction enzyme (Fermentas). T allele undigested and produce 385-bp, while G allele digested and produce 164-bp and 221-bp (Figure 2). The results of PCR-RFLP were 100% concordance with results obtained by T-ARMS-PCR.

### 3.1. Ethical issues

1) The research followed the tenets of the Declaration of Helsinki; 2) informed consent was obtained, and they were free to leave the study at any time; and 3) the research was approved by the ethical committee of Zahedan University of Medical Sciences (#7032).

### 3.2. Statistical analysis

Statistical analyses were done by statistical package SPSS 22. The data were analyzed using χ^2^ test or independent sample t test according to the data. Odds ratio (OR) and 95% CI was computed from unconditional logistic regression analysis to estimate the possible association between the variant and the risk of NS. A *P* value < 0.05 was considered to be statistically significant.

## 4. Results

In this study 138 children with NS and 150 healthy age and sex matched children were enrolled in the study (Table 1). Genotype and allele frequencies of ET-1 rs5370 G/T polymorphism are shown in Table 2. The findings showed that rs5370 variant was not associated with NS in codominant, dominant and recessive inheritance model tested (Table 2). We performed subgroup analysis by sex and the results indicated that the genotype and allelic frequencies of rs5370 polymorphism were not significantly different in male and female (Table 3).

**Table 1:** Demographic and biochemical characteristics of subjects enrolled in the study.

**Parameter**	**Case** **n=138**	**Control** **n=150**	**P**
Sex (M/F)	72/66	74/76	0.706
Age (y)	5.9 ± 3.1	5.8 ± 2.3	0.639
Total protein (g/dL)	4.61 ± 0.90	-	-
Serum albumin (g/dL)	2.37 ± 0.55	-	-
Triglyceride (mg/dL)	317.76 ± 193.33	-	-
Cholesterol (mg/dL)	381.69 ± 125.69	-	-

**Table 2:** Genotype and allele frequency of endothelin 1 rs5370 G/T (lys198Asn) polymorphism in patients with nephrotic syndrome and control subjects

**rs5370 G/T**	**Case** **No. (%)**	**Control** **No. (%)**	**OR (95% CI)**	**P**
Codominant				
GG	50 (36.2)	47 (31.3)	1.00	-
GT	74 (53.7)	89 (59.4)	0.78 (0.47-1.29)	0.369
TT	14 (10.1)	14 (9.3)	0.94 (0.41-2.18)	0.922
Dominant				
GG	50 (36.2)	47 (31.3)	1.00	-
GT+TT	88 (63.8)	103 (68.7)	0.80 (0.49-1.31)	0.386
Recessive				
GG+GT	124 (89.9)	136 (90.7)	1.00	-
TT	14 (10.1)	14 (9.3)	1.10 (0.50-2.39)	0.845
Allele				
G	174 (63.0)	183 (61.0)	1.00	-
T	102 (37.0)	117 (39.0)	0.92 (0.65-1.28)	0.668

**Table 3:** Analysis of the association of the ED-1 rs5370 G>T polymorphism in males and females

**rs5370 G/T**	**Male**		**Female**	
	**Case** **No. (%)**	**Control** **No. (%)**	**Case** **No. (%)**	**Control** **No. (%)**
GG	27 (37.5)	27 (36.5)	23 (34.8	20 (26.3)
GT	37 (51.4)	37 (50.0)	37 (56.1)	52 (68.4)
TT	8 (11.1)	10 (13.5)	6 (9.1)	4 (5.3)
	χ^2^= 0.229,	*P* = 0.891	χ^2^= 2.445,	*P* = 0.294
Allele				
G	91 (63.2)	91 (61.5)	83 (62.9)	92 (60.5)
T	53 (36.8)	57 (38.5)	49 (37.1)	60 (39.5)
	χ^2^= 0.091,	*P* = 0.763	χ^2^= 0.165,	*P* = 0.407

The biochemical parameters of NS patients were analyzed according to the ED-1 rs5370 G/T genotypes and the findings showed that the genotype were not associated with cholesterol, triglyceride, total protein and albumin levels (Table 4).

**Table 4:** Biochemical parameters in NS patients according to the ET-1 rs5370 G/T genotypes

**Parameters**	**ET-1 rs5370 G/T**			**P**
	**GG**	**GT**	**TT**	
Cholesterol (mg/dL)	389.1 ± 148.2	376.5 ± 117.9	385.6 ± 99.1	0.896
Triglyceride (mg/dL)	277.5 ± 123.8	344.5 ± 221.8	303.4 ± 203.7	0.271
Total protein (g/dL)	4.7 ± 0.9	4.6 ± 0.9	4.4 ± 0.9	0.475
Albumin (g/dL)	2.5 ± .0.5	2.3 ± 0.5	2.4 ± 0.9	0.383

## 5. Discussion

NS is a common kidney disease in childhood. In the current study we examined the impact of ET-1 rs5370 variant on NS in childhood. Our findings showed that ET-1 polymorphism was not associated with the risk of NS in our population. Furthermore, this variant was not associated with cholesterol, triglyceride, and total protein and albumin levels.

Yang et al (16) have found that ET-1 rs5370 variant was not associated with NS at genotype or allele frequencies. Although, Plasma ET-1 levels was significantly higher in NS patients in comparison with healthy children. Plasma cholesterol, a hallmark of NS, was significantly associated with the rs5370 GT genotype.

It has been proposed that genetic variation at the ET-1 has an important effect on glomerular filtration but not on urinary albumin excretion in the nondiabetic general population (20). ET-1 is a potent vasoconstrictor and shows various pharmacological responses. Renal function is influenced by direct and indirect action of endothelin. There is evidence that endothelin has an important role in rat renal postnatal development (21).

ET-1, which acts via the specific receptors ET-A and ET-B, has been implicated in the development of renal scarring.

High levels of ET-1 in plasma and tissue concentrations have been shown to be associated with the development of renal interstitial fibrosis and glomerulosclerosis (22-25). Apart from vasoconstriction, it has been revealed that endothelin has multiple biological functions in nonvascular tissues. The paracrine renal endothelin system contributes to the regulation of renal blood flow, GFR, tubular water and sodium reabsorption and control of renin release (24,26,27). In addition to extracellular matrix protein biosynthesis, ET-1 increases the expression of different genes promoting mesangial glomerular and vascular smooth muscle cells proliferation (28,29). These findings proposed that paracrine endothelin system may be involved in the pathogenesis of glomerulosclerosis. There are mounting evidence showed a correlation between glomerulonephritis/glomerulosclerosis and an activated renal endothelin system (30-32).

## 6. Conclusions

In conclusion, the result of this study showed that ED-1 rs5370 polymorphism was not associated with NS in a sample of Iranian population. Further studied with larger sample sizes and different ethnicities are required to confirm our finding.

## Limitations of the study

There are certain limitations in the present study. One of the limitation of our study is that we examined only one variant of ET-1 gene. The second limitation of this study is that we did not investigate the possible association between ED-1 rs5370 polymorphism and response to treatment in patients with NS.

## Authors’ contribution

MH and SSB designed the study, conducted analysis, interpretation of the data and wrote the manuscript. SA and MR collected the data, performed experimental analysis and interpretation of the data. All authors reviewed and approved the final manuscript.

## Conflicts of interest

The authors declare no conflict of interest.

## Funding/Support

This work was supported by a dissertation grant (M.D thesis of Soheib Aryanezhad) from Zahedan University of Medical Sciences.
